# Five-month comparative efficacy evaluation of three ectoparasiticides against adult cat fleas (*Ctenocephalides felis*), flea egg hatch and emergence, and adult brown dog ticks (*Rhipicephalus sanguineus* sensu lato) on dogs housed outdoors

**DOI:** 10.1007/s00436-014-4262-5

**Published:** 2014-12-30

**Authors:** Marie Varloud, Elizabeth Hodgkins

**Affiliations:** 1 Ceva Santé Animale S. A, 10 Avenue de la Ballastière, 33500 Libourne, France; 2 Ceva Animal Health LLC, 8735 Rosehill Road, Suite 300, Lenexa, KS 66215 USA

**Keywords:** Cat flea, *Ctenocephalides felis*, Brown dog tick, *Rhipicephalus sanguineus* sensu lato, Acaricidal efficacy, Insecticidal efficacy

## Abstract

This study was designed to compare the efficacy of three topical combinations on dogs in outdoor conditions against adult cat fleas (*Ctenocephalides felis*), flea egg hatch and emergence, and against adult brown dog ticks (*Rhipicephalus sanguineus* sensu lato). Treatment was performed on day 0 with a placebo; dinotefuran, pyriproxifen and permethrin (DPP); fipronil and (S)-methoprene (FM) or imidacloprid and permethrin (IP). Dogs (*n* = 32), housed outdoors for 7 months, were treated monthly for four consecutive months (on days 0, 30, 60 and 90) and infested with ~100 unfed adult fleas on days 14, 55, 74, 115 and 150 and with ~50 unfed adult ticks on days 28, 44, 88 and 104. Adult fleas were counted and removed 24 h after infestation. Immediately after flea removal, dogs were reinfested with ~100 new adult fleas 72 h prior to egg collection for up to 48 h. Flea eggs were incubated for 32 days, and newly emerged adults were counted. Ticks were counted and removed 48 h after each infestation. FM had >90 % efficacy against fleas at each time point and variable efficacy against ticks (38.0–99.6 %). Efficacy of IP was <90 % against fleas at day 64 and against ticks at day 30 of the first post-treatment. No flea eggs were laid in the treated groups until infestation was carried out >60 days after the last treatment. Despite challenging weather conditions, DPP was highly effective, providing >90 % efficacy against adult ticks as well as adult and immature fleas at every time point of the study.

## Introduction

Fleas and ticks are common and widespread blood-feeding ectoparasites which afflict dogs and cats. The cat flea *Ctenocephalides felis* is a prolific insect, and its population is mostly constituted by immature stages that infest the animal’s environment. Regular and challenging infestations can easily be established from visiting and infested animals. On the animal, the adult cat flea can impair welfare of the host by gradual irritation and pruritus which can turn into flea bite hypersensitivity. It is also a competent vector for numerous diseases, including potentially zoonotic organisms such as *Dipylidium caninum* (Pugh 2001), *Rickettsia felis* (Wedincamp and Foil, 2000) or *Bartonella* spp. (Bouhsira et al. 2012a). The brown dog tick *Riphicephalus sanguineus* sensu lato is a monotropic three-host tick able to perform each stage on dogs, even indoors (Dantas-Torres 2010). It is widely distributed worldwide and within North America. In addition to causing blood loss and irritation, this tick is identified as a vector for different pathogens such as filaroids (*Cercopithifilaria* (Otranto et al. 2012)), protozoa (*Babesia* (Liebisch and Gillani 1979)) and bacteria (*Ehrlichia canis* (Aguiar et al. 2007; Wikswo et al. 2007)). *R sanguineus* is also suspected of being involved in the transmission of *Hepatozoon canis* (Nordgren and Craig 1984), *Anaplasma platys* (Ramos et al. 2014), *Rickettsia* (Trotta et al. 2012), *Bartonella* (Wikswo et al. 2007) and *Coxiella burnetii* (Bernasconi et al. 2002). These pathogens can also be transmitted to humans. To protect companion animals from infestations by these parasites and to reduce the risk of zoonotic transmission, several ectoparasiticide products are available. Most of them are spot-on combinations of insecticide (dinotefuran, fipronil, imidacloprid) and acaricide (fipronil) or acaricide and repellent (permethrin) actives. Some of them also include an insect growth regulator (IGR, such as pyriproxyfen and (S)-methoprene) which provides control of the immature stages of fleas. Despite similar functions or actives, the products differ widely from each other through their formulation. This is based on chemistry, excipients and quality of ingredients and confers upon the products a large part of their efficacy properties (Endris et al. 2003). In active dogs, these properties can make important differences in the final protection provided. Indeed, even companion dogs spend a large part of their time outdoors (Slater et al. 2008) where they may encounter higher numbers of parasites. Because of climatic conditions (rain and UV light), time spent outdoors also increases the risk of removal or inactivation of the topical treatment and potentially reduces the level and duration of efficacy on these animals (Raveton et al. 2006; Schmahl et al. 2009). In order to assess the efficacy of three different spot-on ectoparasiticides for active dogs, their efficacy against fleas and ticks was compared on dogs for 5 months in outdoor conditions.

## Materials and methods

The products were not administered to test animals by any individual involved in performing the post-treatment assessments and observations. Study groups were coded to blind the assessors.

### Animals

**Fig. 1:**
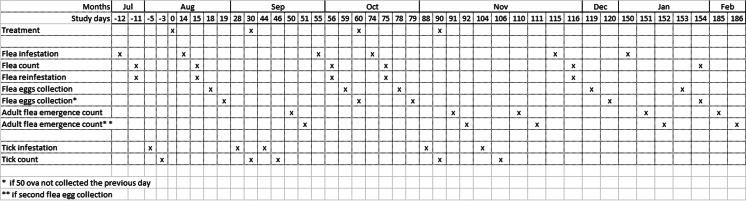
Experimental design

Forty-two healthy adult Beagle dogs with short hair started the 15-day acclimation period. To be enrolled, the dogs should not have been treated with any ectoparasiticide for at least 3 months before the start of the study. The dogs were individually identified by a uniquely numbered tattoo and were fed commercial dog food once daily with water available ad libitum. During the acclimation period, the dogs were bathed with a non-insecticidal shampoo and infested with fleas. Counts were conducted to establish their flea retention level (Fig. 1). Seven dogs with the lowest flea counts and three dogs randomly selected were eliminated from the study and returned to the laboratory colony to reach the objective number of 32 dogs. The selected dogs, 17 males and 15 females, weighed from 10.6 to 16.1 kg and were from 1 to 9 years of age. They were housed individually in indoor pens during the pre-infestation and count period and for 5 days each month during the study for flea ova collection as well as for tick and flea infestation and counting. During the rest of the study period, the dogs were housed in compatible same sex pairs with dogs from the same treatment group in outdoor runs. The runs were designed to expose dogs to the outdoor environment but also enabled them to seek refuge from sunlight and rain, thus providing shelter at their pleasure. The last two allocated dogs were an intact male and a female that were separated in the pen with individual shelters. The general health of all dogs was observed once daily during the study. On each treatment day, clinical observations of all dogs were conducted at approximately 1, 2, 3 and 4 h post-treatment. This study plan was approved by the ethics committee of Nu-Era Farms.

### Allocation

Allocation to treatment groups was carried out on day 2. The 32 selected dogs were ranked, in descending order of day 2 body weights, into eight replicates of four animals each. Each dog within each replicate was randomly assigned, without regard to sex, to one of the four treatment groups (1, 2, 3 and 4). Each group consisted of eight dogs. Each dog was also randomly assigned to an indoor/outdoor run. Both male and female dogs were represented in each treatment group.

### Treatment application

Each dog was treated with the allocated treatment on days 0, 30, 60 and 90 of the study (Table 1, Fig. 1). Dogs in group 1 received the placebo control solution (CS) containing the vehicle of the commercial formulation Vectra 3D® (Ceva Animal Health LLC Lenexa, KS, USA). Dogs in group 2 were treated with the commercial formulation Vectra 3D® (DPP) containing dinotefuran (4.95 % *w/w*), pyriproxifen (0.44 %) and permethrin (36.08 %). Group 3 was treated with the commercial formulation Frontline Plus® (Merial Ltd., Duluth, GA) containing fipronil (9.8 %) and (S)-methoprene (8.8 %) (FM), and group 4 was treated with the commercial formulation K9 Advantix® 55 (Bayer HealthCare, Shawnee Mission, KS, USA) containing imidacloprid (8.8 %) and permethrin (44.0 %) (IP). The commercial formulations were administered topically, as spot-on, in accordance with the manufacturers’ label directions. CS and DPP were administered at a rate of 3.6 mL per dog applied equally (1.2 mL per site) in three spots at the shoulder blades, mid-back and base of tail. FM was administered at a rate of 1.34 mL per dog, in one spot applied at the shoulder blades. IP was administered at a rate of 2.5 mL per dog, in three equal spots between the shoulder blades, mid-back and base of the tail.

**Table 1:** Study design

Study steps	Study days					
Treatment administration	0	30	60	90		
Flea infestation	−12	14	55	74	115	150
Flea count	−11	15	56	75	116	154
Flea reinfestation		15	56	75	116	
Flea eggs collection	18	59	78	119	153	
Flea eggs collection^a^	19	60	79	120	154	
Adult flea emergence count	50	91	110	151	185	
Adult flea emergence count^b^	51	92	111	152	186	
Tick infestation	−5	28	44	88	104	
Tick count	−3	30	46	90	106	

^a^If 50 ova not collected the previous day

^b^If second flea egg collection performed

### Flea and tick challenge

The laboratory colonies of fleas and ticks used in the study were not known to be resistant to any pesticide, and both the fleas and ticks had been in culture for less than 3 years. All dogs were infested with 100 unfed *C. felis* adult fleas on days 12 (during acclimation), 14, 55, 74, 115 and 150 of the study. Flea counts were conducted 24 h after infestation except for the final infestation when flea counts were conducted 96 h post-infestation (on day 154). For counts, the hair coat on all parts of the dog including the tail was examined in a methodical manner so that fleas were combed off the dogs and live fleas were counted. Following the 24-h flea counts, all dogs were reinfested with new fleas (~100) on days −11, 15, 56, 75 and 116 for flea egg collection. Flea eggs were collected over a period of 48 h beginning 3 days after infestation, on egg collection liners placed under the indoor run resting areas. The liners were removed after overnight egg collection and carefully swept to collect the debris and eggs that had fallen from the animal. Following egg collection, the fleas were combed off the dogs but no counts were conducted. For each dog, available eggs (up to 50, if available) were counted and placed in dishes holding groups of 25 eggs per dish. The eggs were incubated with culture media for 4 days and examined to determine larval hatch. The dishes were held under the same conditions for an additional 28 days to determine the number of emerged adults.

All dogs were infested with 50 unfed *R. sanguineus* adult ticks (male:female ratio of 1:1) on days 5 (during acclimation), 28, 44, 88 and 104 of the study. Tick counts were conducted approximately 48 h after infestation (days 30, 46, 90 and 106) by systematically examining all areas of the animal. When performing the counts, the animal’s hair was pushed against its nape so that the skin and the ticks were exposed. Ticks were removed with blunt pointed forceps and placed in a dish of alcohol for counting.

### Statistical analysis

#### Adult flea and tick effectiveness

To determine efficacy against adult fleas and ticks, the counts were transformed to the natural logarithm of (count + 1) to calculate geometric means (GM). Percent efficacy for each treatment group on each flea or tick count day was calculated using the following equation:

$$ \%\ \mathrm{Efficacy} = \left(\frac{\mathrm{Geometric}\kern0.5em \mathrm{Mean}\ \mathrm{Count}\ \mathrm{Control}\ \mathrm{Group} - \mathrm{Geometric}\kern0.5em \mathrm{Mean}\ \mathrm{Count}\ \mathrm{Treated}\ \mathrm{Group}}{\mathrm{Geometric}\kern0.5em \mathrm{Mean}\ \mathrm{Count}\ \mathrm{Control}\ \mathrm{Group}}\right)*100 $$

The treatments were compared using a *t* test for means with poolable variances or for means with unequal variances, as appropriate. Variances were compared using an F test, and Satterthwaite’s approximation was used to determine the degree of freedom for the unequal variance tests; when one variance was 0, the variance was unequal by definition, and when both variances were 0, no comparisons were possible. Each treated group was compared to the CS group, and the group treated with DPP was compared to groups treated with FM and IP. Statistical significance was declared at a two-sided *p* value of 0.05.

#### Ovicidal and adult flea emergence

Percentage hatch and adult emergence from flea eggs were transformed to the arcsine (radians) of the square root of the proportion to calculate means. Mean angle was then back transformed. This procedure maintained non-missing values for dogs participating in the study. Percent efficacy for each treatment group on each day was calculated using the following equation:

$$ \%\ \mathrm{Efficacy} = \left(\frac{\mathrm{Retransformed}\ \mathrm{Mean}\ \mathrm{of}\ \mathrm{Control}\kern0.5em \mathrm{Group} - \mathrm{Retransformed}\ \mathrm{Mean}\ \mathrm{of}\ \mathrm{Treated}\ \mathrm{Group}}{\mathrm{Retransformed}\ \mathrm{Mean}\ \mathrm{of}\ \mathrm{Control}\ \mathrm{Group}}\right)*100 $$

The data were analyzed as described above for adult fleas and ticks. All analyses and calculations were performed using SAS Version 9.2 (SAS Institute, Cary, NC).

### Guidelines

This study was carried out at Nu-Era Farms (Stillwater, OK, USA) in compliance with Good Clinical Practice requirements (VICH GL9, May 2001). In addition, the study was conducted in compliance with US EPA Product Performance Test Guidelines OPPTS 810.3300: Treatments to Control Pests of Humans and Pets.

## Results

**Fig. 2:**
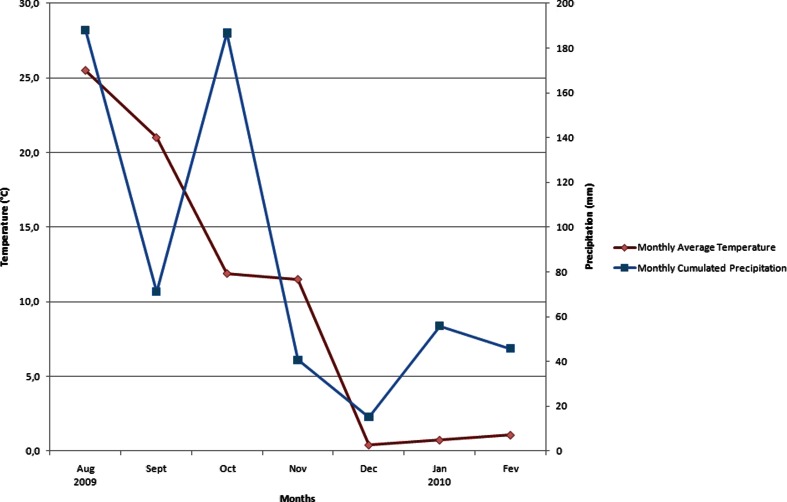
Climatological data of Stillwater, Oklahoma (US) between August 2009 and February 2010 (National Climatic Data Center)

No adverse effects to any of the treatment applications were observed in any dogs during the study. The climatic conditions in the location for the study duration were highly variable (Fig. 2).

### Efficacy against adult fleas (*Ctenocephalides felis*)

Pre-treatment GM flea counts ranged from 67.4 to 69.0 for all study groups. During the study, GM flea counts ranged from 67.4 to 81.6 for the CS group (Table 2).

**Table 2:** Geometric mean number of fleas (*Ctenocephalides felis*) per dog and the percent efficacy based on geometric mean flea counts for groups treated with either CS, DPP, FM or IP days 0, 30, 60 and 90 of the study

		Geometric mean number of fleas/dog^a^				% Efficacy		
Study day	Days since last treatment^b^	CS	DPP	FM	IP	DPP	FM	IP
−11	NA	67.4	67.5	68.4	69.0	NA	NA	NA
15	15	76.3	1.1^*^	0.3^*^	1.1^*^	98.6	99.7	98.6
56	26	74.5	1.3^*^	0.7^*^	1.6^*^	98.3	99.1	97.9
75	15	81.6	0.1^*^	0.0^*^	0′0^*^	99.9	100	100
116	26	76.2	0.2^*^	0.5^*^	0.9^*^	99.8	99.4	98.9
154	64	76.4	6.3^*^	1.2^*^	8.4^*^	91.7	98.4	89.1

*CS* control solution, *DPP* dinotefuran, pyriproxifen and permethrin, *FM* fipronil and (S)-methoprene, *IP* imidacloprid and permethrin, *NA* not applicable

*Geometric mean flea counts significantly different from the CS group (*p* < 0.01)

^a^Eight dogs in each group, flea counts conducted 24 h post-infestation

^b^Last or the most recent treatment

For dogs treated with DPP, FM or IP, efficacies ranged from 98.3 to 99.9 %, 99.1 to 100 % and 97.9 to 100 %, respectively, when counts were conducted 15 or 26 days after treatments and were 91.7, 98.4 and 89.1 %, respectively, when counts were conducted 64 days after the last treatment. Compared to the CS group, the three treated groups had lower (*p* < 0.005) flea counts. When the DPP-treated group was compared to the FM- or IP-treated groups, it was not possible to detect any difference in efficacy at any of the assessment time points.

### Efficacy against flea (*Ctenocephalides felis*) egg hatch and emerging adults

After the additional flea challenges on days 15, 56, 75 and 116 of the study, no eggs were available for collection on days 18–19, 59–60, 78–79 and 119–120 from all three treated groups. After the last challenge on day 150, eggs were collected from all groups on days 153–154 of the study (Table 3).

**Table 3:** Percent egg hatch and adult flea emergence with percent efficacies against *Ctenocephalides felis* for groups treated with CS DPP, FM or IP on days 0, 30, 60 and 90 of the study

		Egg hatch						
Egg collection		% hatch				% efficacy		
Study days	Days since last treatment	CS	DPP	FM	IP	DPP	FM	IP
18–19	18–19	78.1	na^*^	na^*^	na^*^	na	na	na
59–60	29–30	74.6	na^*^	na ^*^	na^*^	na	na	na
78–79	18–19	75.1	na^*^	na^*^	na	na	na	na
119–120	29–30	80.7	na^*^	na^*^	na^*^	na	na	na
153–154	63–64	79.6	1.0^*^	0.2^*^	33.7^*+^	98.7	99.7	57.7
		Adult flea emergence						
Egg collection		% emergence				% efficacy		
Study days	Days since last treatment	CS	DPP	FM	IP	DPP	FM	IP
18–19	18–19	74.3	na^*^	na^*^	na^*^	na	na	na
59–60	29–30	73.9	na^*^	na^*^	na^*^	na	na	na
78–79	18–19	74.8	na^*^	na^*^	na^*^	na	na	na
119–120	29–30	80.4	na^*^	na^*^	na^*^	na	na	na
153–154	63–64	78.3	1.0^*^	0.1^*^	26.5^*+^	98.7	99.9	66.2

*CS* control solution, *DPP* dinotefuran, pyriproxifen and permethrin, *FM* fipronil and (S)-methoprene, *IP* imidacloprid and permethrin, *na* not applicable because no egg could be collected for incubation

*Significantly different from the CS group (*p* < 0.01)

+Significantly different from the DPP group (*p* < 0.01)

Except for two animals in the FM-treated group, flea eggs were collected from every animal in all four study groups on days 153–154 of the study (63–64 days after the last treatment). This measurement was added to measure the residual activity of all formulations. At this time point, for dogs treated with DPP, FM and IP, efficacy against egg hatch was 98.7, 99.7 and 57.7 %, respectively, and the efficacy against adult flea emergence was 98.7, 99.9 and 62.2 %, respectively. When compared, the CS group had a much higher (*p* < 0.0001) percentage of egg hatch and adult flea emergence than the DPP- and FM-treated groups. The CS group had a higher percentage of egg hatch (*p* = 0.0019) and of adult emergence (*p* = 0.0005) than the IP-treated group. The DPP-treated group had a lower percentage of egg hatch (*p* = 0.0012) and adult flea emergence (*p* = 0.0024) than the IP-treated group, but no difference was detected between the DPP-treated group and the FM-treated group.

### Efficacy against adult ticks (*R. Sanguineus*)

Pre-treatment GM tick counts for all study groups ranged from 19.5 to 21.9. For the CS group, GM tick counts ranged from 20.1 to 23.2 during the study (Table 4).

**Table 4:** Mean number of ticks (*Riphicephalus sanguineus*) per dog and the percent efficacy based on geometric mean tick counts for groups treated with CS, DPP, FM or IP on days 0, 30, 60 and 90 of the study

		Geometric mean number of tick/dog^a^				% efficacy		
Study day	Days since last treatment^b^	CS	DPP	FM	IP	DPP	FM	IP
3	NA	20.1	21.9	19.5	21.9	NA	NA	NA
30	30	21.8	1.5^*^	13.5^*+^	4.1^*^	93.2	38.0	81.4
46	16	22.5	0.4^*^	0.1^*^	0.3^*^	98.4	99.6	98.9
90	30	20.1	0.9^*^	10.5^*+^	0.3^*^	95.3	47.7	98.6
106	16	23.2	1.7^*^	3.3^*^	0.6^*^	92.6	85.8	97.4

^a^Eight dogs in each group, tick counts conducted 48 h post-infestation

^b^Last or the most recent treatment

*CS* control solution, *DPP* dinotefuran, pyriproxifen and permethrin, *FM* fipronil and (S)-methoprene, *IP* imidacloprid and permethrin, *NA* not applicable

*Geometric mean tick count significantly different from the CS (*p* < 0.01)

+Significantly different from the DPP group (*p* < 0.01)

For dogs treated with the DPP formulation, efficacies ranged from 92.6 to 98.4 % when tick counts were conducted 16 or 30 days after the most recent treatments. For FM-treated dogs, the tick efficacies ranged from 85.8 to 99.6 % for counts conducted 16 days after treatment and from 38.0 to 47.7 % for counts conducted 30 days after treatment. For the IP-treated dogs, the efficacies were between 97.4 to 98.9 % and 81.8 to 98.9 % when counts were conducted 16 or 30 days after the initial or the most recent treatments, respectively.

Whatever the time point, the CS group had higher (*p* < 0.01) tick counts compared to any treated group. The DPP-treated group had lower tick counts than the FM group when counts were conducted 30 days after treatment (*p* = 0.0034 at day 30 and *p* = 0.0015 at day 90). It was not possible to detect any difference in tick counts between the DPP- and IP-treated groups at any of the assessment time points.

## Discussion

### Methodological considerations

The dogs spent 113 of the 171 days (66 %) of study duration after first treatment application, in outdoor housing. Their housing conditions can be considered as representative of a free-outdoor access. The study was run between 22 July 2009 and 8 February 2010 in Oklahoma (USA). One treatment was performed on the 6 August 2009, during one of the rainiest (188 mm of precipitation) and hottest (25.5 °C) months of the study (Fig. 2).

As demonstrated in the CS group, flea and tick challenges were successful during the whole duration of the study. Moreover, at the beginning of the experiment and before administration of the treatments, the four groups exhibited very similar levels of GM flea (from 67.4 to 69.0) and tick (from 19.5 to 21.9) counts.

In this study, infestations by the two parasites were artificial: the fleas and ticks were directly deposited on the haircoat of dogs, whereas in natural conditions, fleas jump on their targeted host and questing ticks crawl from down to upside positions. When they infest a permethrin-treated host, these parasites are repelled and leave the host. While the present experiment was designed to assess insecticidal and acaricidal efficacy, it was not designed to assess the repellency properties of IP and DPP permethrin-based combinations.

### Efficacy against adult fleas

When efficacy was assessed 15 or 26 days after treatment, the three products exhibited very high adulticidal efficacy levels (>97.9–100 %). Because of these asymptotic levels, it was not possible to detect any difference between the products since all of them demonstrated a much lower (*p* < 0.0001) adult flea count than the control. We can therefore consider them all suitable for flea adulticidal activity in outdoor dogs. In a previous field study, FM and DPP formulations already provided very similar levels of protection against fleas (Dryden et al. 2011).

Interestingly, adulticidal efficacy was assessed at the end of the study, 64 days after the last treatment, mimicking a lack of compliance. At this time point, again, all the groups treated with actives had lower adult flea counts than the CS group. The different treatments provided a 2-month protection against artificial infestations with adult fleas on dogs, without any negative interaction of outdoor housing conditions.

It is generally considered that permethrin (at 50 mg/Kg BW), one of the adulticidal actives of DPP, is deposited in the upper layers of the *stratum corneum* and on the surface of hairs (Lüssenhop et al. 2012). Fipronil, however, spreads across the body within the skin’s sebum and accumulates in pilo-sebaceous glands and in superficial layers of the epidermis. This was demonstrated, in Beagle dogs, with a volume and dose of product (FM, 1.34 mL, 10 mg/Kg) similar to the one used in our study (Cochet et al. 1997). However, 2 months after treatment, in outdoor conditions, this property of FM did not confer any detectable advantage to this formulation as compared to DPP regarding flea adulticidal activity.

### Efficacy against immature stages of fleas

For all DPP, FM and IP treatment groups and collection points, no eggs were collected up to day 120 of the study. This is probably the consequence of the high adulticidal efficacy of the products which killed the fleas before they laid their eggs as the animals were treated either 18–19 or 29–30 days before collection of eggs. Because no adult flea count was performed after egg collection, this hypothesis cannot be fully verified but the flea count conducted following day 115 infestation highly suggests this. Indeed, we counted only zero to one flea (six dogs without fleas) in the DPP-treated group, zero to three fleas (four dogs without fleas) in the FM-treated group and zero to nine fleas (five dogs without fleas) in the IP-treated group. Because of this high adulticidal activity, the adult flea population and the subsequent egg production may therefore have been highly reduced, falling under our detection limit.

In order to reduce the interaction between adulticidal and IGR actives, egg collection and subsequent evaluation of egg hatch and adult emergence were assessed 64 days after the last treatment. Despite the high adulticidal efficacy (>97.9 %), the number of flea-free dogs dropped to two, three and zero for DPP, FM and IP, respectively, and eggs were collected in every group. DPP and FM formulations contain a true IGR active: pyriproxyfen and (S)-methoprene, respectively, whereas IP has none. This resulted in a higher viability of the eggs, with larvae hatching more successfully in the IP-treated group than in the DPP- and FM-treated groups. The larvae were also more successful in their pupation and emergence process up to adults in the IP-treated group than in the DPP- and FM-treated groups. Both FM and DPP provided very satisfactory levels of protection against immature stages of fleas. These results confirmed previous observations with DPP performed on transplanted and ready to hatch fleas (Bouhsira et al. 2012b).

An increasing number of products without IGR actives is available to pet owners. These results demonstrate that such products cannot provide the same duration of protection against the immature population of fleas. Moreover, this experimental design was favourable to IGR-like activity. Indeed, it only mimicked the control of offspring production from fleas infesting the treated pets at specific time points. These fleas were directly exposed to the treated pets. Unlike in real situations, neither environmental infestation (wild animals, etc.) nor egg infestation of the dog surroundings occurred. In more realistic circumstances, more benefits of true IGR substances, which also prevent fleas from developing in the environment of the treated pets, would be expected.

### Efficacy against ticks (*R. sanguineus*)

Despite the free outdoor access housing of the dogs, the tick counts were lower in all treated groups compared to the CS group. However, the different formulations provided different levels of protection. DPP, containing the acaricide permethrin, had higher efficacy than FM, containing the acaricide fipronil, as measured 30 days after the first (93.2 vs. 38.0 %) and third (95.3 vs. 47.7 %) monthly treatments. At these two time points, all the dogs treated with FM had at least 6 ticks attached and, more precisely, 7 to 16 ticks after the first treatment and 6 to 20 ticks after the third treatment. Moreover, a strong reduction in acaricidal efficacy was detected between the 16th (99.6–85.8 %) and the 30th (38.0–47.7 %) day after treatment in the FM-treated group. This phenomenon is compatible with a reduced residual concentration of the acaricide-active fipronil in the coat of the animals at the end of the expected treatment duration (1 month). In the outdoor conditions of our experiment, the spreading properties of the fipronil-based formulation did not provide any advantage as compared to the permethrin-based formulations. This difference in acaricidal activity between these fipronil-based and permethrin-based products has been documented in several studies: a laboratory experiment in dogs infested with *R. sanguineus* (Dryden et al. 2006), a multi-centre field study against adult *Ixodes* and *Rhipicephalus* ticks (Hellmann et al. 2003) and under natural conditions against both immature and adult stages of *R. sanguineus* (Otranto et al. 2005). These experiments all demonstrated that, at the end of the treatment duration, permethrin-based products such as IP and DPP are more effective against *R. sanguineus* than the fipronil-based combination FM. However, the residual acaricidal efficacy of the IP group was only 81.4 % 30 days after the first treatment.

DPP was already shown to be highly effective against *Amblyomma americanum* and *A. maculatum* (Coyne 2009). Our study confirms that DPP provides a satisfactory level of protection to dogs against *R. sanguineus*, without any adverse effect and despite the fact that dogs spent 5 months in challenging a free access outdoor housing. It can therefore be concluded that DPP is appropriate to protect active dogs with outdoor access against *R. sanguineus* ticks.

## Conclusions

This experiment demonstrated that, under free outdoor-access housing, dogs treated with FM or DPP were protected against all stages of fleas for 2 months following four monthly applications. However, IP efficacy did not remain above 90 % 30 days after administration. The study also confirmed that fipronil-based formulations such as FM are not optimal for the control of artificial infestation by *R. sanguineus* ticks in dogs, especially with free outdoor access. Natural infestations can be even more challenging, with average tick loads already reported to reach 150 ticks per dog (Brianti et al. 2010). In the circumstances of this study, only permethrin-based formulations provided a satisfactory range of efficacy. However, with only 81.4 % efficacy, the IP formulation did not provide the expected minimum 90 % efficacy against ticks 30 days after the first treatment. DPP was therefore the only formulation providing both satisfactory efficacy levels against all stages of fleas (*C. felis*) and against ticks (*R. sanguineus*) on dogs housed with free outdoor access.
